# Supplementary material for: Ligand Screening and Discovery using Cocktail Soaking and Automated MicroED
Source: bioRxiv. 2025 Feb 20:2025.02.18.638921. Preprint. [Version 1] doi: 10.1101/2025.02.18.638921 (PMC11870483; doi:10.1101/2025.02.18.638921)
Supplement: Supplement 1 [file media-1.pdf]

Supporting Information:

Ligand Screening and Discovery using Cocktail Soaking and Automated MicroED

Jieye Lin[1], Marc J. Gallenito[1], Johan Hattne[1,3] and Tamir Gonen[1,2,3]*

[1] Department of Biological Chemistry, University of California, Los Angeles, 615 Charles E. Young Drive South, Los Angeles, California 90095, United States

[2] Department of Physiology, University of California, Los Angeles, 615 Charles E. Young Drive South, Los Angeles, California 90095, United States

[3] Howard Hughes Medical Institute, University of California, Los Angeles, Los Angeles, California 90095, United States

[*] Corresponding Author T.G. tgonen@g.ucla.edu

Table S1. Data processing and model refinement statistics for **TLN-1**.

Name of the protein[a]	**TLN-1**	**TLN-1**	**TLN-1**
Set/Grid[b]	A1	A2	B
Accelerating Voltage (kV)	300	300	300
Wavelength (Å)	0.0197	0.0197	0.0197
Resolution range (Å)	38.99 - 2.001 (2.08 - 2.0)	44.44 - 2.599 (2.86 - 2.6)	40.06 - 2.58 (2.84 - 2.58)
Space group	P 6_1 2 2	P 6_1 2 2	P 6_1 2 2
Unit cell parameters (Å,°)	94.66 94.66 132.96 90 90 120	94.32 94.32 132.77 90 90 120	92.51 92.51 128.72 90 90 120
Total reflections	290748	131162	140415
Unique reflections	24393	11334	10822
Multiplicity	11.92	11.57	12.97
Completeness (%)	99.7	99.9	99.9
I/sigma (I)	3.25	3.12	3.74
R-meas	0.576	0.616	0.583
$CC_{1/2}$	0.943	0.927	0.969
Wilson B-factor	21.38	32.15	35.36
Reflections used in refinement (#)	24316	11285	10776
Reflections used for R-free (#)	1218	564	540
R-work	0.2565	0.2549	0.2100
R-free	0.2761	0.3114	0.2490
Number of non-hydrogen atoms (#)	2527	2489	2507
macromolecules	2469	2469	2469
ligands	5	5	5
solvent	53	15	33
Protein residues (#)	317	317	317
RMS (bonds)	0.004	0.002	0.002
RMS (angles)	0.63	0.44	0.50
Ramachandran favored (%)	96.50	95.86	95.22
Ramachandran allowed (%)	3.50	3.82	4.46
Ramachandran outliers (%)	0.00	0.32	0.32
Rotamer outliers (%)	1.19	0.40	0.00
Clashscore	2.51	2.72	3.77
Average B-factor	17.79	20.20	26.49
macromolecules	17.79	20.21	26.58
ligands	18.08	19.36	27.16
solvent	17.89	19.10	19.92

Notes: [a]Thermolysin was abbreviated as "**TLN**". [b]Data collected from experimental sets/grids A1-B, see Table 1.

Table S2. Data processing and model refinement statistics for **TLN-2**.

Name of the protein[a]	**TLN-2**	**TLN-2**
Set/Grid[b]	A2	C
Accelerating Voltage (kV)	300	300
Wavelength (Å)	0.0197	0.0197
Resolution range (Å)	47.48 - 2.35 (2.53 - 2.35)	44.54 - 2.77 (3.17 - 2.77)
Space group	P 6_1 2 2	P 6_1 2 2
Unit cell parameters (Å,°)	94.96 94.96 133.45 90 90 120	94.44 94.44 134.22 90 90 120
Total reflections	179080	81213
Unique reflections	15416	9526
Multiplicity	11.62	8.53
Completeness (%)	99.7	99.6
I/sigma (I)	2.09	2.24
R-meas	0.755	0.660
$CC_{1/2}$	0.911	0.900
Wilson B-factor	31.93	33.45
Reflections used in refinement (#)	15373	9491
Reflections used for R-free (#)	769	474
R-work	0.2731	0.2577
R-free	0.3014	0.2984
Number of non-hydrogen atoms (#)	2484	2468
macromolecules	2432	2432
ligands	22	22
solvent	30	14
Protein residues (#)	316	316
RMS (bonds)	0.003	0.002
RMS (angles)	0.49	0.47
Ramachandran favored (%)	96.18	96.18
Ramachandran allowed (%)	3.82	3.82
Ramachandran outliers (%)	0.00	0.00
Rotamer outliers (%)	0.40	1.59
Clashscore	2.53	1.90
Average B-factor	26.75	18.52
macromolecules	26.76	18.47
ligands	27.87	27.27
solvent	25.45	13.12

Notes: [a]Thermolysin was abbreviated as “**TLN**”. [b]Data collected from experimental sets/grids A2 and C, see Table 1.

Table S3. Data processing and model refinement statistics for **TLN-3**.

Name of the protein[a]	**TLN-3**	**TLN-3**
Set/Grid[b]	A2	D
Accelerating Voltage (kV)	300	300
Wavelength (Å)	0.0197	0.0197
Resolution range (Å)	39.24 - 2.896 (3.32 - 2.9)	47.34 - 2.23 (2.37 - 2.23)
Space group	$P\ 6_1\ 2\ 2$	$P\ 6_1\ 2\ 2$
Unit cell parameters (Å,°)	94.7 94.7 135.03 90 90 120	94.68 94.68 131.66 90 90 120
Total reflections	87163	236138
Unique reflections	7131	17629
Multiplicity	12.22	13.39
Completeness (%)	83.8	99.7
I/sigma (I)	2.39	2.16
R-meas	0.666	0.733
$CC_{1/2}$	0.887	0.940
Wilson B-factor	44.43	36.08
Reflections used in refinement (#)	7112	17580
Reflections used for R-free (#)	356	881
R-work	0.2890	0.2628
R-free	0.3298	0.2760
Number of non-hydrogen atoms (#)	2453	2494
macromolecules	2432	2432
ligands	12	12
solvent	9	50
Protein residues (#)	316	316
RMS (bonds)	0.002	0.003
RMS (angles)	0.43	0.51
Ramachandran favored (%)	93.95	95.86
Ramachandran allowed (%)	5.73	4.14
Ramachandran outliers (%)	0.32	0.00
Rotamer outliers (%)	0.79	1.59
Clashscore	2.75	2.97
Average B-factor	27.13	32.68
macromolecules	27.16	32.81
ligands	31.41	41.53
solvent	15.03	24.57

Notes: [a]Thermolysin was abbreviated as "**TLN**". [b]Data collected from experimental sets/grids A2 and D, see Table 1.

Table S4. Data processing and model refinement statistics for **PK-4**

Name of the protein[a]	**PK-4**	**PK-4**
Set/Grid[b]	E	F
Accelerating Voltage (kV)	300	300
Wavelength (Å)	0.0197	0.0197
Resolution range (Å)	43.27 - 2.02 (2.18 - 2.02)	47.72 - 2.25 (2.48 - 2.25)
Space group	$P\ 4_3\ 2_1\ 2$	$P\ 4_3\ 2_1\ 2$
Unit cell parameters (Å,°)	67.42 67.42 103.12 90 90 90	67.49 67.49 105.89 90 90 90
Total reflections	128645	112273
Unique reflections	14035	12043
Multiplicity	9.17	9.32
Completeness (%)	86.2	98.5
I/sigma (I)	3.72	4.56
R-meas	0.457	0.488
$CC_{1/2}$	0.955	0.974
Wilson B-factor	23.63	32.09
Reflections used in refinement (#)	13959	12022
Reflections used for R-free (#)	698	601
R-work	0.2601	0.2127
R-free	0.3146	0.2721
Number of non-hydrogen atoms (#)	2075	2069
macromolecules	2029	2029
ligands	18	14
solvent	28	26
Protein residues (#)	279	279
RMS (bonds)	0.002	0.003
RMS (angles)	0.45	0.52
Ramachandran favored (%)	97.11	96.03
Ramachandran allowed (%)	2.89	3.97
Ramachandran outliers (%)	0.00	0.00
Rotamer outliers (%)	0.00	0.94
Clashscore	3.01	4.27
Average B-factor	18.40	26.11
macromolecules	18.34	26.03
ligands	25.72	41.87
solvent	18.47	23.54

Notes: [a]Proteinase K was abbreviated as "**PK**". [b]Data collected from experimental sets/grids E and F, see Table 1.

Table S5. Data processing and model refinement statistics for **PK-5**

Name of the protein[a]	**PK-5**	**PK-5**
Set/Grid[b]	E	G
Accelerating Voltage (kV)	300	300
Wavelength (Å)	0.0197	0.0197
Resolution range (Å)	40.74 - 1.97 (2.09 - 1.97)	47.55 - 1.97 (2.09 - 1.97)
Space group	$P\ 4_3\ 2_1\ 2$	$P\ 4_3\ 2_1\ 2$
Unit cell parameters (Å,°)	67.51 67.51 102.17 90 90 90	67.25 67.25 101.31 90 90 90
Total reflections	137334	139116
Unique reflections	17208	17111
Multiplicity	7.98	8.13
Completeness (%)	98.9	99.9
I/sigma (I)	3.35	4.23
R-meas	0.518	0.460
$CC_{1/2}$	0.962	0.978
Wilson B-factor	22.65	28.98
Reflections used in refinement (#)	17174	17042
Reflections used for R-free (#)	859	851
R-work	0.2341	0.2016
R-free	0.2838	0.2315
Number of non-hydrogen atoms (#)	2082	2111
macromolecules	2029	2029
ligands	16	16
solvent	37	66
Protein residues (#)	279	279
RMS (bonds)	0.007	0.006
RMS (angles)	0.80	0.78
Ramachandran favored (%)	97.83	97.11
Ramachandran allowed (%)	2.17	2.89
Ramachandran outliers (%)	0.00	0.00
Rotamer outliers (%)	0.00	0.00
Clashscore	7.02	4.51
Average B-factor	18.90	24.93
macromolecules	18.85	24.87
ligands	26.57	34.36
solvent	18.17	24.21

Notes: [a]Proteinase K was abbreviated as “**PK**”. [b]Data collected from experimental sets/grids E and G, see Table 1.

Table S6. Data processing and model refinement statistics for **PK-6**

Name of the protein[a]	**PK-6**	**PK-6**
Set/Grid[b]	E	H
Accelerating Voltage (kV)	300	300
Wavelength (Å)	0.0197	0.0197
Resolution range (Å)	34.7 - 1.801 (1.88 - 1.8)	43.33 - 2.3 (2.53 - 2.3)
Space group	$P\ 4_3\ 2_1\ 2$	$P\ 4_3\ 2_1\ 2$
Unit cell parameters (Å,°)	67.46 67.46 101.12 90 90 90	67.68 67.68 102.11 90 90 90
Total reflections	177311	99068
Unique reflections	22253	11100
Multiplicity	7.97	8.93
Completeness (%)	99.6	99.6
I/sigma (I)	3.95	3.25
R-meas	0.413	0.531
$CC_{1/2}$	0.974	0.932
Wilson B-factor	20.1	27.2
Reflections used in refinement (#)	22226	11050
Reflections used for R-free (#)	1112	555
R-work	0.2269	0.2463
R-free	0.2574	0.2943
Number of non-hydrogen atoms (#)	2147	2101
macromolecules	2029	2029
ligands	43	44
solvent	75	28
Protein residues (#)	279	279
RMS (bonds)	0.006	0.002
RMS (angles)	0.80	0.46
Ramachandran favored (%)	97.83	94.95
Ramachandran allowed (%)	1.81	5.05
Ramachandran outliers (%)	0.36	0.00
Rotamer outliers (%)	0.94	0.00
Clashscore	5.49	2.25
Average B-factor	17.91	20.00
macromolecules	17.78	19.87
ligands	24.70	26.46
solvent	17.55	19.57

Notes: [a]Proteinase K was abbreviated as “**PK**”. [b]Data collected from experimental sets/grids E and H, see Table 1.

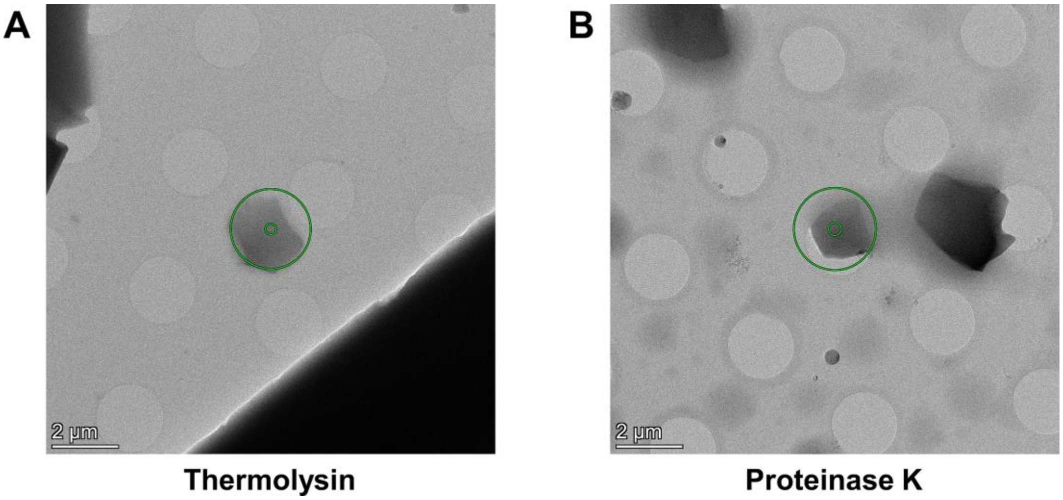

Figure S1. Representative images of microcrystals of (A) thermolysin and (B) proteinase K under imaging mode (2250 X).

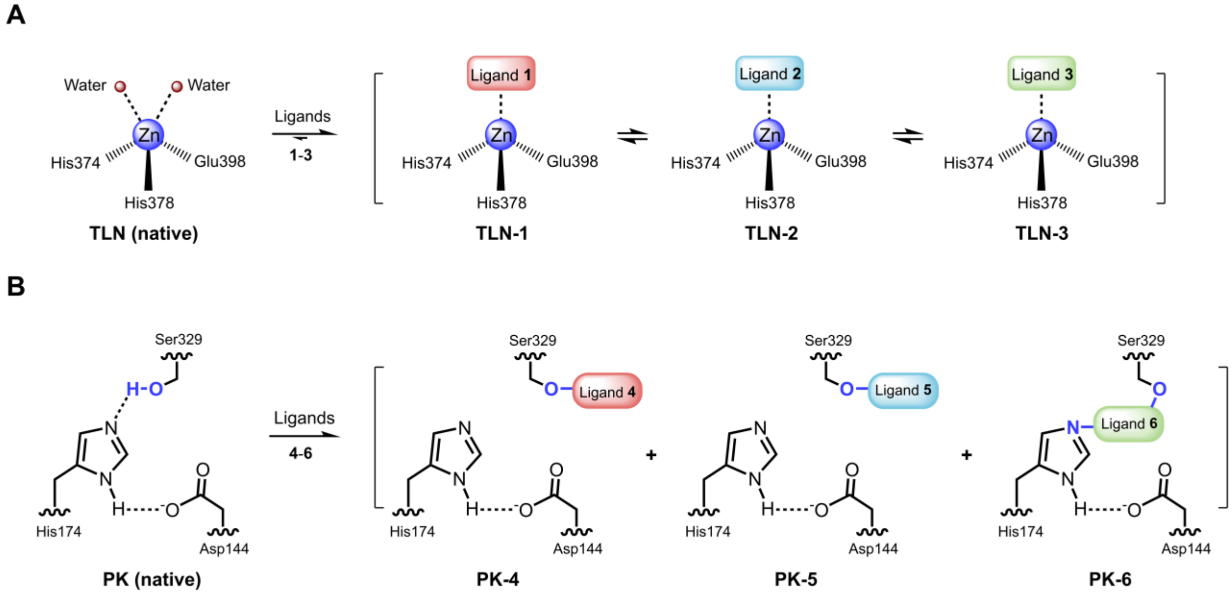

Figure S2. Schemes of protein-ligand binding mechanisms in cocktail soaking of (A) thermolysin with ligands **1-3** and (B) proteinase K with ligands **4-6**. Thermolysin was abbreviated as “**TLN**”, proteinase K was abbreviated as “**PK**”.

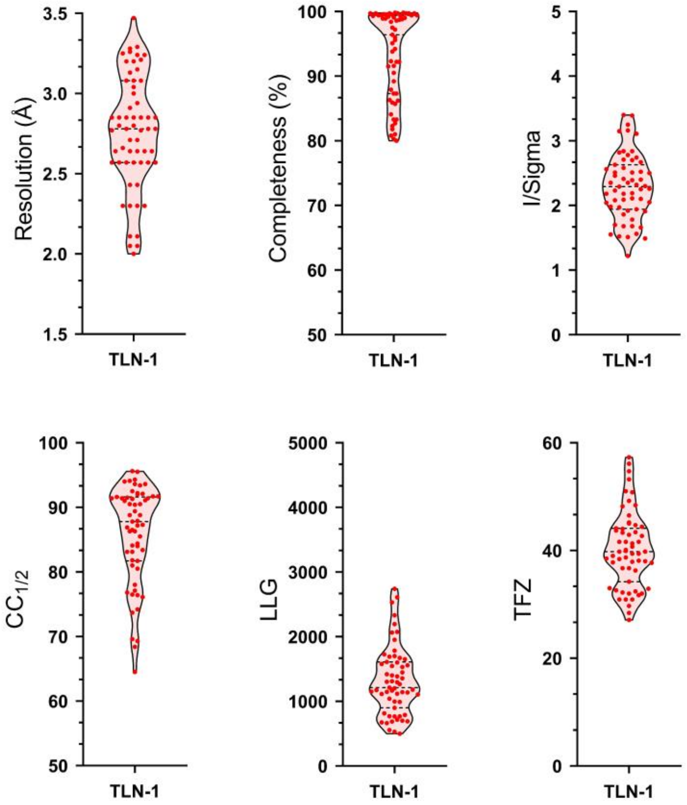

Figure S3. Statistics of **TLN-1** determined from cocktail soaking set A1. Thermolysin was abbreviated as “**TLN**”.

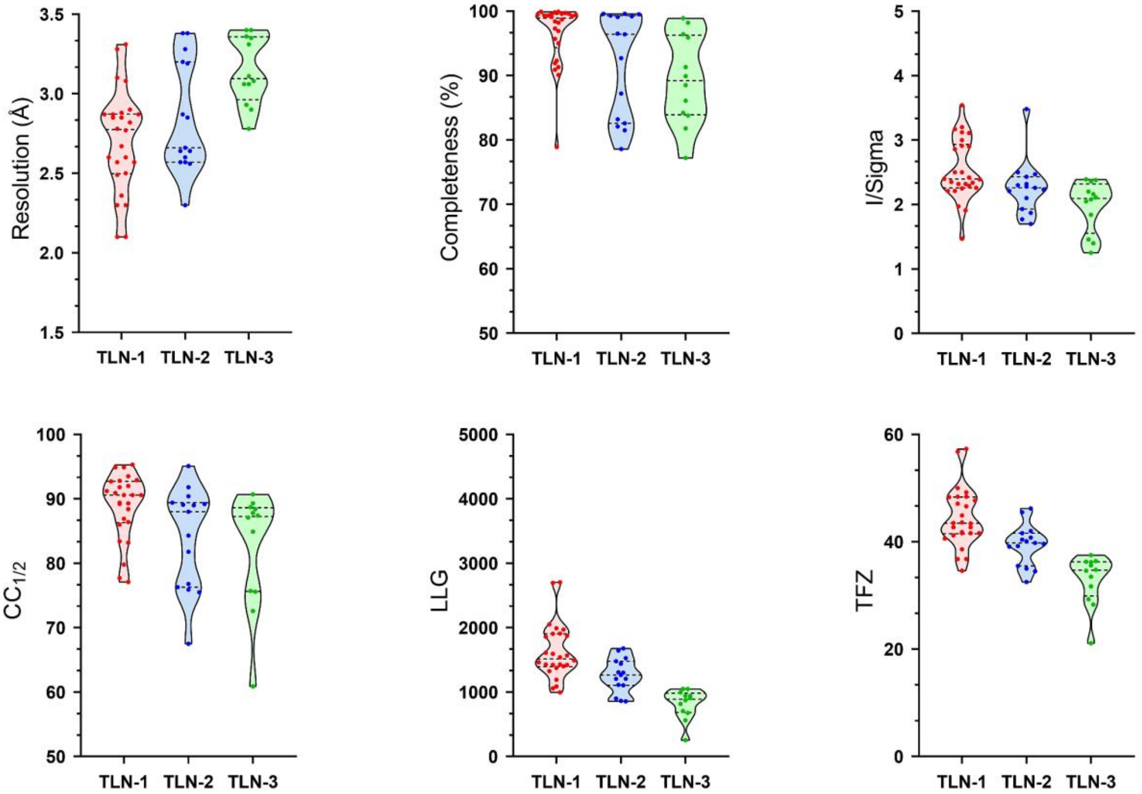

Figure S4. Statistics of **TLN-1/2/3** determined from cocktail soaking set A2. Thermolysin was abbreviated as “**TLN**”.

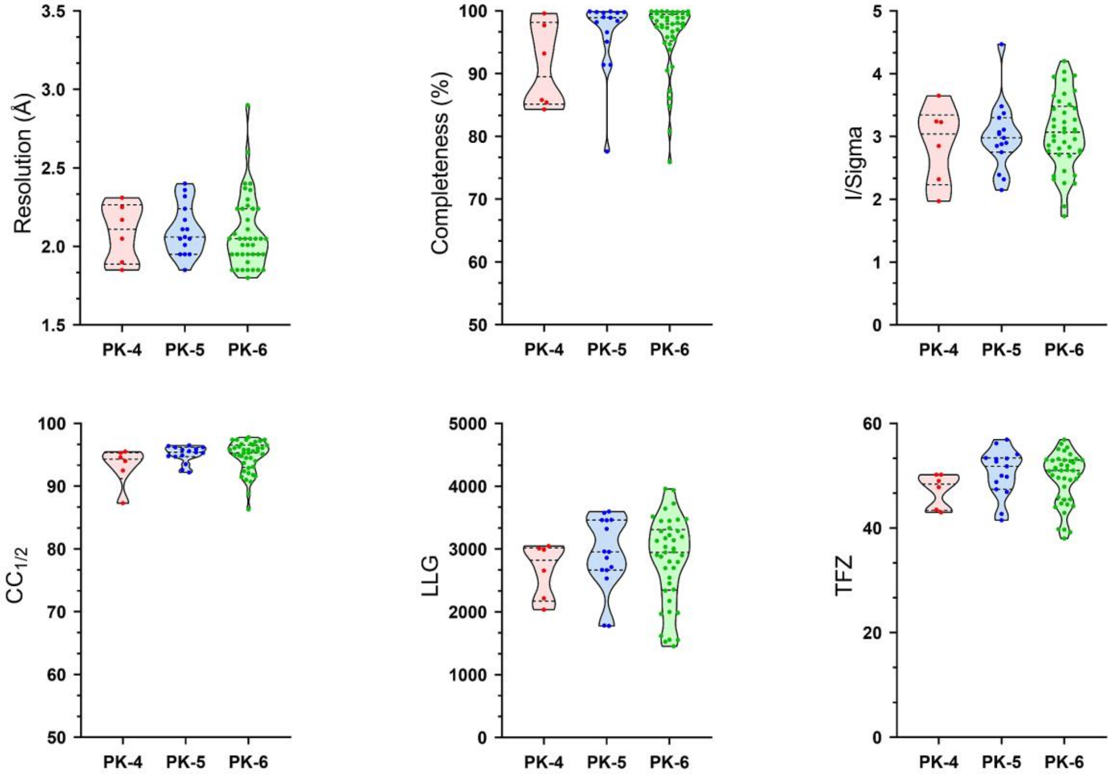

Figure S5. Statistics of **PK-4**/**5**/**6** determined from cocktail soaking set E. Proteinase K was abbreviated as "**PK**".

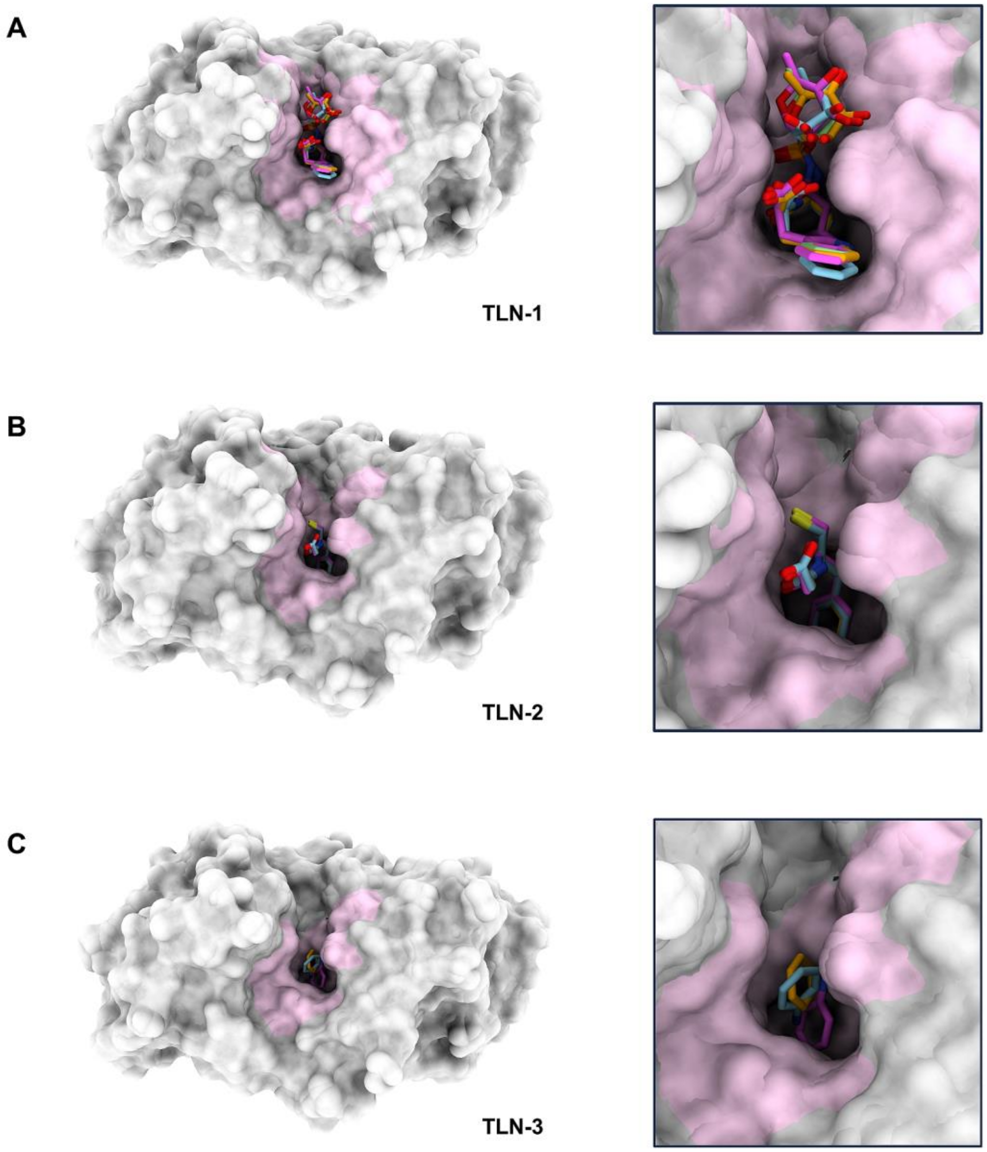

Figure S6. Overlay of **TLN-1/2/3** structures from different soaking sets and literature.[1-3] (A) **TLN-1** were colored by different sets: set A1, in orange; set A2, in blue; set B, in green; 1TLP, in magenta. (B) **TLN-2** were colored by different sets: set A2, in orange; set C, in blue; 1Z9G, in magenta. (C) **TLN-3** were colored by different sets: set A2, in orange; set D, in blue; 3MS3, in magenta. See Table 1 for details. Thermolysin was abbreviated as "**TLN**".

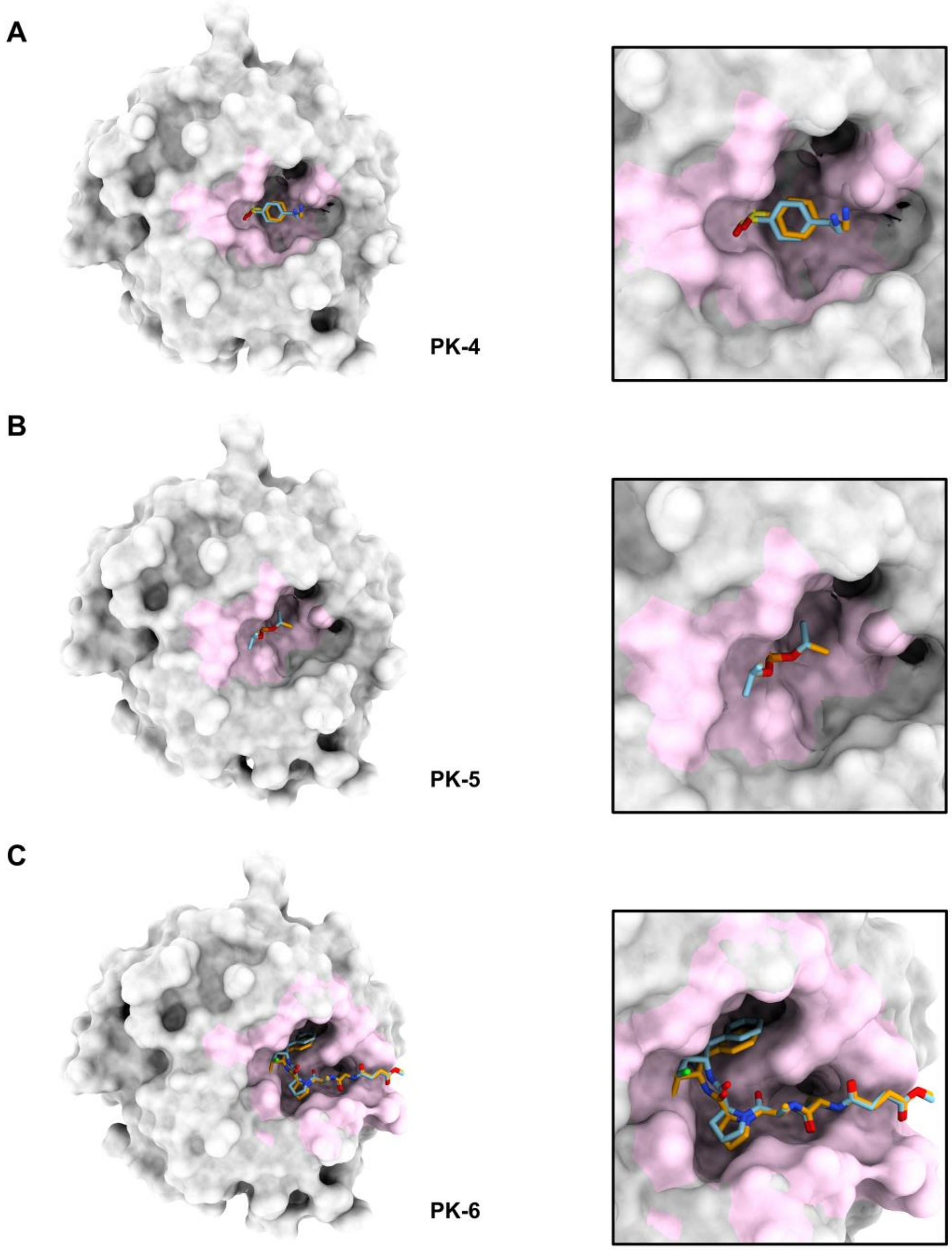

Figure S6. Overlay of **PK-4**/**5**/**6** structures from different soaking sets. (A) **PK-4** were colored by different sets: set E, in orange; set F, in blue. (B) **PK-5** were colored by different sets: set E, in orange; set G, in blue. (C) **PK-6** were colored by different sets: set E, in orange; set H, in blue. See Table 1 for details. proteinase K was abbreviated as “**PK**”.

Reference

1 D. E. Tronrud, A. F. Monzingo, B. W. Matthews, *Eur. J. Biochem.* **1986**, *157*, 261-268.
2 S. L. Roderick, M. C. Fournie-Zaluski, B. P. Roques, B. W. Matthews, *Biochemistry* **1989**, *28*, 1493-1497.
3 J. Behnen, H. Köster, G. Neudert, T. Craan, A. Heine, G. Klebe, *ChemMedChem* **2012**, *7*, 248-261.
